# Supplementary material for: ProteinShader: illustrative rendering of macromolecules
Source: BMC Struct Biol. 2009 Mar 30;9:19. doi: 10.1186/1472-6807-9-19 (PMC2672931; doi:10.1186/1472-6807-9-19)
Supplement: Additional file 1 — ProteinShader program without source code. This compressed file contains the complete ProteinShader program including associated libraries, but no source code. A README.txt file gives an overview of the ProteinShader distribution, and the index.html file in the help subdirectory has directions on getting started with the program as well as a set of tutorials. [file 1472-6807-9-19-S1.zip › ProteinShader-beta-0_9_4-binary/help/api/org/proteinshader/gui/listeners/controlpanel/VisibilityPanelListenerFactory.html]

VisibilityPanelListenerFactory (ProteinShader API)


|  |  |  |
| --- | --- | --- |
| **Overview** \| **Package** \| **Class** \| **Use** \| **Tree** \| **Deprecated** \| **Index** \| **Help** | |  |
| **PREV CLASS**   NEXT CLASS | **FRAMES**    **NO FRAMES**     **All Classes** |  |
| SUMMARY: NESTED \| FIELD \| CONSTR \| METHOD | DETAIL: FIELD \| CONSTR \| METHOD |  |


---


## org.proteinshader.gui.listeners.controlpanel Class VisibilityPanelListenerFactory

```
java.lang.Object
  org.proteinshader.gui.listeners.controlpanel.VisibilityPanelListenerFactory
```

---

``` public class VisibilityPanelListenerFactory extends Object ```

Creates the listener for the VisibilityPanel.

---

**Constructor Summary**

`VisibilityPanelListenerFactory(Mediator mediator,
VisibilityPanel visibilityPanel)`
  
          Constructs a VisibilityPanelListenerFactory.


| **Method Summary** | |
| --- | --- |
| `ChangeListener` | `createAlphaSliderChangeListener()`             Creates a change listener for the alpha slider. |
| `ActionListener` | `createInvisibleButtonActionListener()`             Creates an action listener for the "Invisible" button. |
| `ActionListener` | `createOpaqueButtonActionListener()`             Creates an action listener for the "Opaque" button. |
| `ActionListener` | `createTranslucentButtonActionListener()`             Creates an action listener for the "Translucent" button. |

**Methods inherited from class java.lang.Object**

`clone, equals, finalize, getClass, hashCode, notify, notifyAll, toString, wait, wait, wait`

**Constructor Detail**

### VisibilityPanelListenerFactory

```
public VisibilityPanelListenerFactory(Mediator mediator,
                                      VisibilityPanel visibilityPanel)
```

:   Constructs a VisibilityPanelListenerFactory.

    **Parameters:**: `mediator` - the centralized Mediator object that listeners call on to accomplish their task.: `visibilityPanel` - the VisibilityPanel that this class creates listeners for.


**Method Detail**

### createOpaqueButtonActionListener

```
public ActionListener createOpaqueButtonActionListener()
```

:   Creates an action listener for the "Opaque" button. When the
    action listener is called, it will apply VisibilityEnum.OPAQUE and
    an alpha value of 1.0 to whatever item or items are selected in
    the radio panel.

    :   **Returns:**: The action listener.

---


### createInvisibleButtonActionListener

```
public ActionListener createInvisibleButtonActionListener()
```

:   Creates an action listener for the "Invisible" button. When the
    action listener is called, it will apply VisibilityEnum.INVISIBLE
    and an alpha value of 0.0 to whatever item or items are selected
    in the radio panel.

    :   **Returns:**: The action listener.

---


### createTranslucentButtonActionListener

```
public ActionListener createTranslucentButtonActionListener()
```

:   Creates an action listener for the "Translucent" button.
    When the action listener is called, it will apply
    VisibilityEnum.TRANSLUCENT and an alpha value of
    VisibilityPanel.SLIDER\_INIT to whatever item or items
    are selected in the radio panel.

    :   **Returns:**: The action listener.

---


### createAlphaSliderChangeListener

```
public ChangeListener createAlphaSliderChangeListener()
```

:   Creates a change listener for the alpha slider. When the change
    listener is called, it will update the "% translucent" text field,
    calculate the corresponding alpha value, and then apply that alpha
    value to the item or items currently selected in the RadioPanel.

    :   **Returns:**: The change listener.


---


|  |  |  |
| --- | --- | --- |
| **Overview** \| **Package** \| **Class** \| **Use** \| **Tree** \| **Deprecated** \| **Index** \| **Help** | |  |
| **PREV CLASS**   NEXT CLASS | **FRAMES**    **NO FRAMES**     **All Classes** |  |
| SUMMARY: NESTED \| FIELD \| CONSTR \| METHOD | DETAIL: FIELD \| CONSTR \| METHOD |  |


---

# *Copyright © 2007-2008*
